# Coronavirus HKU1 in Children, Brazil, 1995

**DOI:** 10.3201/eid1706.101381

**Published:** 2011-06

**Authors:** Luiz G. Góes, Edison L. Durigon, Angélica A. Campos, Noely Hein, Saulo D. Passos, José A. Jerez

**Affiliations:** Author affiliations: Universidade de São Paulo, São Paulo, Brazil (L.G. Góes, E.L. Durigon, A.A. Campos, N. Hein, J.A. Jerez);; Faculdade de Medicina de Jundiai, Jundiai, Brazil (S.D. Passos)

**Keywords:** HKU1, human coronavirus, acute respiratory infection, respiratory viruses, viruses, children, Brazil, letter

**To the Editor**: Coronavirus HKU1 is a newly identified human coronavirus (HCoV) that was reported first in 2005 in Hong Kong Special Administrative Region, People’s Republic of China; later in Australia, Europe, and the United States, and more recently in Brazil, demonstrating a global distribution (*1**–**3*). We examined the circulation of HCoV in Brazil and the possible presence of the new HCoV types, with special attention to coronavirus HKU1, in samples collected back to 1995, tested by using universal coronavirus PCR.

The epidemiologic profile of HCoV was retrospectively investigated with samples collected during March–December 1995 in a pediatric ward of University Hospital, São Paulo University, São Paulo, Brazil. The Ethics Committee on Research Involving Human Subjects of the Institute of Biomedical Sciences, University of São Paulo, approved the study. Samples of nasopharyngeal aspirates were collected from 169 hospitalized children, ages 7 days–15 years, of whom 104 had respiratory symptoms, 23 had enteric disease, and 3 had both (*4*). The mean age of the study population was 19.6 months (median 7 months). Viral nucleic acid was extracted from specimens by using Trizol (Invitrogen, Carlsbad, CA, USA) following the manufacturer’s instructions. Total RNA was then submitted to reverse transcription PCR with a High-Capacity cDNA Archive Kit (Applied Biosystems, Foster City, CA, USA) by using random primers according to the manufacturer’s instructions. The cDNA obtained was screened with primers able to amplify a 220-bp product in the conserved polymerase region of all known HCoVs (*5*) and other coronaviruses (e.g., bovine coronavirus). We used cDNA obtained from cultured human rectal tumor cell line HRT-18G cells inoculated with bovine coronavirus strain Kakegawa as positive controls for PCR.

In an attempt to improve the sensitivity of HKU1 detection, we analyzed samples with negative results by PCR with a nested PCR specific for coronavirus HKU1. This nested assay was designed on an alignment of our HKU1-positive sample sequence (BRA169) and different HKU1 genotype sequences deposited in GenBank. Primers Fn-HKU1 (forward 5′-CGTGCYATG CCAAATATTTTGCG-3′, HKU1-NC_006577, nt 15433–15454) and Rn-HKU1 (reverse 5′-TAGCAACCGCCACACATAAC-3′, HKU1-NC_006577, nt 15562–15581) produced an amplicon of 149 bp. The nested PCR was run in a 50-µL reaction comprising 10 µL of PCR product, 1.5 units of DNA Polymerase (Biotools, Madrid, Spain), 1 µmol/L of each primer, 200 µmol/L of each dNTP (Applied Biosystems), 2 mmol/L MgCl_2_, and 1× buffer. PCR mixtures were heated to 95°C for 5 min, followed by 35 cycles of 1 min at 95°C, 30 s at 62°C, and 40 s at 72°C, followed by a final 10 min at 72°C. Clinical samples positive for HKU1 were used as positive controls in the HKU1 nested PCR. All fragments obtained from PCR and nested PCR were analyzed in a 2% (wt/vol) agarose gel by electrophoresis, stained with 0.5 µg/mL of ethidium bromide, and subsequently sequenced to confirm the type of coronavirus. Nucleotide sequencing reactions were performed on both amplicon strands by using an ABI PRISM Big Dye Cycle Sequencing Kit with the ABI PRISM 3100 automatic sequencer (Applied Biosystems).

Six (3.6%) samples tested positive for HCoV-HKU1: 2 samples by PCR and 4 by nested PCR. HCoV types 229E, OC43, and NL63 were not detected in any sample by PCR. Samples positive for HCoV were associated with pertussis, pneumonia, bronchiolitis, and diarrhea (Table).

**Table:** Epidemiologic and laboratory data of children with coronavirus infection, Brazil, 1995*

Specimen no.: HCoV strain by pol analyses	Age/sex	Sample collection date	Clinical diagnosis	Co-infections	Detection method, fragment sequenced
09: HKU1A	3 mo/F	Mar	Pertussis	ND	Nested PCR, 143 bp
37: HKU1A	2 mo/M	Apr	Bronchiolitis plus bronchopneumonia	RSV	Nested PCR, 143 bp
90: HKU1B	4 mo/M	Jul	Upper respiratory infection	ND	Nested PCR, 143 bp
99: HKU1 B	9 y/M	Jul	Pleural effusion pneumonia	ND	Nested PCR, 143 bp
104: HKU1 A	2 mo/F	Jul	Pertussis	ND	Pancoronavirus PCR, 143 bp
169: HKU1B	3 y/F	Nov	Fever, diarrhea	Worms	Pancoronavirus PCR, 173 bp

*HCoV, human coronavirus; ND, not detected; RSV, respiratory syncytial virus.

In a recent review, an analysis of 18 studies indicated that the median (range) incidence of HCoV-HKU1 was 0.9% (0%–4.4%) (*2*), which is similar to the detection rate in our study. To our knowledge, the only study that has screened for HKU1 in Brazil found that 0.48% of children were positive for HKU1 (*3*), which is lower than our results.

Although we did not detect other HCoV types, all HCoV types were detected previously in Brazil in samples collected during 2006–2008 (*3**,**6*). The absence of detection of 229E, OC43, and NL63 HCoV might have resulted from the seasonality and natural viral year cycle or from the characteristics of the children studied because we included samples from children hospitalized with or without respiratory disease.

BLAST search (www.ncbi.nlm.nih.gov/blast/Blast.cgi) and phylogenetic analysis of amplicons from PCR and nested PCR indicated that samples were positive for HKU1 genotype B (samples BRA169, BRA90, and BRA99) or HKU1 genotype A (samples BRA09, BRA37, and BRA104). The sequences obtained in this study have been deposited in GenBank under accession nos. FJ931534.1 (BRA169), GU904424 (BRA37), GU904427 (BRA104), GU904423 (BRA09), GU904425 (BRA90), and GU904426 (BRA99).

This may be the oldest collection of human samples in which HKU1 has been detected. To our knowledge, the oldest previous sample positive for HCoV-HKU1 was detected in children in Finland during 1996–1998, without an exact date specified (*7*). Retrospective studies also have been conducted in the United States and Greece that showed the HKU1 virus in different countries in Europe and North America before its discovery (*8**,**9*). We have confirmed the circulation of HKU1 coronaviruses in children in Brazil in 1995.
